# Thinking Outside the Therapeutic Box: The Potential of Polyphenols in Preventing Chemotherapy-Induced Endothelial Dysfunction

**DOI:** 10.3390/cells14080566

**Published:** 2025-04-09

**Authors:** Luke Tillman, Jaume Margalef Rieres, Elena Ahjem, Fynn Bishop-Guest, Meghan McGrath, Helena Hatrick, Md Zahidul Islam Pranjol

**Affiliations:** 1 School of Clinical Medicine, University of Cambridge, Cambridge CB2 0SP, UK; lt544@cam.ac.uk (L.T.); jm2615@cam.ac.uk (J.M.R.); mm2657@cam.ac.uk (M.M.); hrh40@cam.ac.uk (H.H.); 2 School of Life Sciences, University of Sussex, Falmer, Brighton BN1 9QG, UK

**Keywords:** polyphenols, endothelium, cancer, chemotherapy, cardiovascular disease, endothelial dysfunction

## Abstract

The numerous side effects and adverse health implications associated with chemotherapies have long plagued the field of cancer care. Whilst in some cases a curative measure, this highly toxic intervention consistently scores poorly on quantitative measures of tolerability and safety. Of these side effects, cardiac and microvascular defects pose the greatest health risk and are the leading cause of death amongst cancer survivors who do not succumb to relapse. In fact, in many low-grade cancers, the risk of recurrence is far outweighed by the cardiovascular risk of morbidity. As such, there is a pressing need to improve outcomes within these populations. Polyphenols are a group of naturally occurring metabolites that have shown potential vasoprotective effects. Studies suggest they possess antioxidant and anti-inflammatory activities, in addition to directly modulating vascular signalling pathways and gene expression. Leveraging these properties may help counteract the vascular toxicity induced by chemotherapy. In this review, we outline the main mechanisms by which the endothelium is damaged by chemotherapeutic agents and discuss the ability of polyphenols to counteract such side effects. We suggest future considerations that may help overcome some of the published limitations of these compounds that have stalled their clinical success. Finally, we briefly explore their pharmacological properties and how novel approaches could enhance their efficacy while minimising treatment-related side effects.

## 1. Introduction

**Figure 1:**
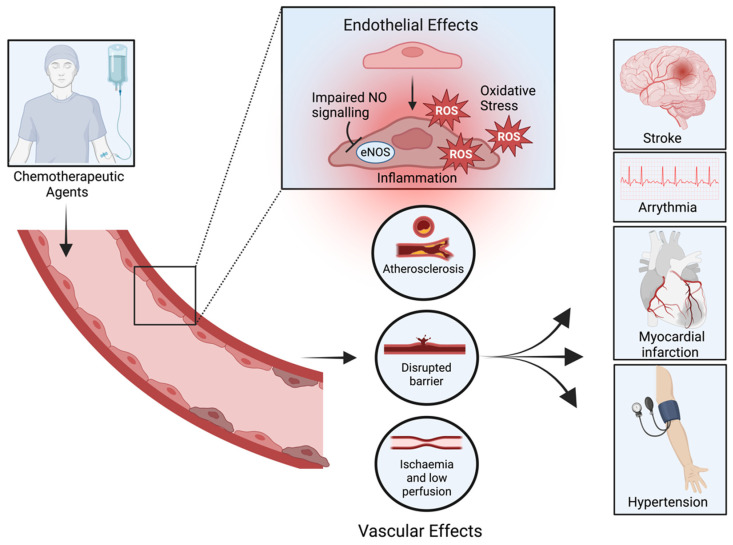
Chemotherapy mechanisms of cardiotoxicity. Impaired NO signalling, oxidative stress, and inflammation caused by chemotherapy-induced vascular damage contribute to atherosclerosis, capillary disruption, ischemia, and reduced perfusion, ultimately leading to phenotypic effects resembling myocardial infarction and stroke.

Cancer is the second-greatest contributor to global mortality [1], with a staggering 28 million new cases per annum estimated by 2040 [2]. Thanks to significant therapeutic advancements over the past two decades, cancer survival statistics have vastly improved [3,4]. Owing to its low cost and ease of distribution, chemotherapy remains the first-line treatment for almost all cancers (Table 1) [5]. Whilst effective at inducing the necrosis of metabolically overactive tumours, chemotherapies are plagued by their off-target side effects, most notably those that impact the cardiac system and vasculature (Figure 1) [6,7,8]. Indeed, the greatest cause of mortality in cancer survivors, outside of malignant recurrence, is cardiovascular disease [9,10,11]. In a cohort of over 3.2 million cancer survivors, cardiovascular mortality was found to be tenfold higher than in the general population after controlling for age, race, and sex [12]. Moreover, breast cancer survivors were found to carry a 170% greater risk of myocardial infarction following treatment with the selective oestrogen receptor modulator, tamoxifen [13].

Previously, research has focused on the direct damage from chemotherapy to cardiomyocytes; however, an emerging area of study is the mechanisms by which chemotherapy damages the vascular endothelium, leading to cardiovascular complications.

Several clinical trials have explored the use of ACE inhibitors, angiotensin receptor–neprilysin inhibitors (ARNIs), and beta blockers in preventing adverse events, but so far none have shown long-term clinical success [21,22]. Thus, further consideration is needed to mitigate these effects for patients receiving cancer care.

Polyphenols are a class of natural compounds that have been shown to implement multiple benefits in both preventing and restoring a dysfunctional endothelium [23,24,25]. It is therefore hypothesised that the administration of polyphenols alongside chemotherapy could mitigate its damaging effects on the vascular endothelium.

## 2. Chemotoxic-Induced Endothelial Dysfunction

Cardiotoxicity refers to a spectrum of disorders that result in damage to the heart and its associated vessels. However, the term is often misunderstood due to the diverse range of organic and inorganic insults to which the heart is exposed. While various imaging, serological, and molecular techniques have been developed to address these complexities, they often rely on arbitrary cut-off values that fail to accurately capture the extent of cardiac damage during chemotherapy [26]. The most widely accepted physiological definition of cardiotoxicity, developed by the cardiac review and evaluation committee during the trastuzumab clinical trials, defines it as any drug-induced reduction in left ventricular function or the onset of heart failure [27]. When applied to the context of chemotherapeutics, this definition is often broadened to include any treatment-related cardiac damage. The ambiguity surrounding this definition has hindered research progress and the development of innovative solutions. Critically, this broad framework does not adequately account for the variety of insults that lead to symptomatic events. For instance, key components of the cardiovascular system, such as the vascular endothelium—which is often the initial point of contact for many chemotherapeutic agents—are frequently overlooked until significant dysfunction has occurred. This limitation underscores the urgent need for more comprehensive and nuanced approaches to evaluating and mitigating cardiotoxicity.

**Figure 2:**
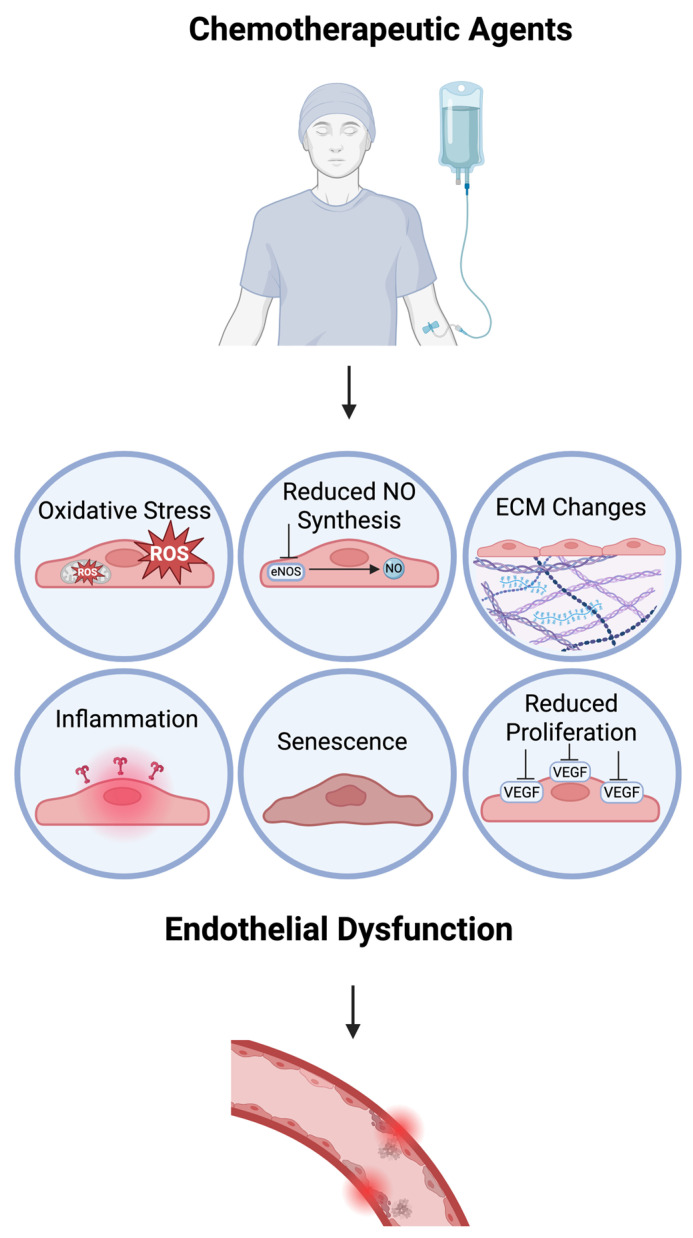
Chemotherapeutic agents can damage endothelial cells and impair function. This occurs through both direct and indirect processes. Core mechanisms include oxidative stress, inflammation and cytokine release, the reduced synthesis of NO due to the inhibition of endothelial eNOS, cellular senescence, reduced proliferation due to VEGF inhibition, and ECM changes.

Disruption to the vascular endothelium has emerged as the primary mechanism by which chemotherapeutic agents contribute to cardiovascular disease [8,28]. At the core of this disruption is endothelial dysfunction (ED), a critical driver of the vascular changes underlying these conditions [29,30]. ED is characterised by a reduction in the synthesis and release of protective elements, leading to harmful effects, including vasoconstriction, platelet aggregation, inflammation, and mitogenic activities (Figure 2) [31,32,33]. Identifying this vascular pathology early is a crucial component in improving outcomes, leading to a surge in research attempts to identify adequate markers of endothelial damage [34,35,36].

The assessment of blood fluid patterns and fluid shear stress in the circulation system has been used as a reliable marker of ED for some time. In response to increased blood flow (shear stress), the endothelium facilitates vasodilation through a critical physiological process known as flow-mediated dilation (FMD). Clinically, FMD is a widely accepted non-invasive measure of endothelial function, in which the percentage change in the arterial lumen diameter is assessed following reactive hyperaemia [37]. Impaired FMD is considered indicative of endothelial dysfunction and has been used to monitor vascular health across a range of disease states [38,39]. This approach has proven particularly valuable in oncology settings, where vascular toxicity is a growing concern. For instance, evidence has robustly supported this notion in children undergoing anthracycline treatment for acute lymphoblastic leukaemia (ALL). In this cohort, the brachial artery FMD was approximately four times lower compared to age- and sex-matched controls when assessed between two months and seven years following the completion of therapy [40]. Notably, the FMD was not influenced by the duration of time since the final anthracycline infusion [40]. Similar data have also been collated in treatment schedules for solid tumours. Anastasiou et al. recently documented a gradual decline in FMD among women with breast cancer from the baseline to one year post-completion of anthracycline-based chemotherapy. Comparing baseline measurements to those taken 12 months post-completion of anthracycline and trastuzumab therapy, the FMD showed a significant reduction (6.95 ± 2.86% vs. 5.03 ± 2.83%, *p* = 0.006) [41]. Thus, the utilisation of FMD as a marker for chemotherapy-induced ED appears highly promising.

In healthy individuals, FMD is primarily driven by the enzymatic production of nitric oxide (NO), a powerful vasodilator essential for maintaining vascular health. However, in pathological conditions characterised by an overproduction of reactive oxygen species (ROS), such as superoxide, NO is rapidly degraded, resulting in impaired FMD [42]. Under these circumstances, vasodilation in larger blood vessels is diminished, leading to tissue ischaemia, atherosclerosis, thrombosis, and other inflammatory processes [43].

Anthracyclines contribute to excessive ROS generation, causing mitochondrial dysfunction and endothelial damage. This surge in ROS significantly decreases the expression and phosphorylation of endothelial nitric oxide synthase (eNOS), thereby reducing eNOS activity and impairing NO production [44]. As a pivotal regulator of vascular homeostasis, eNOS activity is a critical target for both the treatment and prevention of ED. Notably, studies that have successfully enhanced eNOS function through transcriptional or translational approaches have demonstrated considerable preclinical promise [45,46,47,48]. To date, the exact mechanisms underlying the loss of eNOS functionality during chemotherapy remain nuanced; however, its reduction in endothelial cells has been associated with prolonged hypoxia and ischaemia [49,50].

Numerous cases of vascular ischaemia have been attributed to chemotherapeutic agents [51,52,53]. Among these, 5-fluorouracil (5-FU), an antimetabolic pyrimidine analogue widely used as a first-line treatment for colorectal cancers, has emerged as a significant contributor to cardiovascular toxicity [54]. Both 5-FU and its oral pro-drug, capecitabine, are frequently linked to cardiac side effects, particularly myocardial ischemia [55]. Mechanistically, these agents have been shown to induce vascular damage through various pathways. For instance, studies using the vascular endothelial cell model EA.hy926 have demonstrated that both 5-FU and capecitabine promote cellular senescence and the upregulation of the endothelial damage marker CD146, shedding light on their deleterious effects on endothelial integrity [56].

More recently, Hammon et al. revealed in an analysis of 30 patients undergoing 5-FU-based cancer treatment that 5-FU diminishes microvascular reactivity following eNOS-dependent local heating. Using laser Doppler flowmetry, the authors measured the cutaneous red blood cell flux, which was used as an index of cutaneous blood flow [57]. All patients receiving 5-FU displayed a decrease in cutaneous vascular conductance (CVC) comparative to a control group receiving no chemotherapy and presented with a significantly lower resting mean arterial pressure (MAP). CVC was used to account for potential differences and fluctuations in the MAP. Whilst the underlying mechanism for these findings was not fully elucidated, the authors did note a significant decrease in *eNOS* expression in 5-FU-treated human coronary artery endothelial cells [57]. These findings may explain why 5-FU-treated patients have previously shown a statistically significantly higher 6- and 12-month risk for myocardial infarction compared with control subjects [58].

The use of platinum-based chemotherapeutics shows similarly damning statistics regarding cardiovascular health. In a retrospective epidemiological study of more than 15,000 patients, Fung et al. demonstrated a 5-fold increase in the standardised cardiovascular mortality following cisplatin-based chemotherapy that was confined primarily to the first year after treatment [59]. Building on this understanding, Nuver et al. observed that incremental treatment with a cisplatin–bleomycin cocktail (CBC) exacerbated ED in the human dermal microvascular endothelial cell line HMEC-1. CBC exposure led to the upregulation of intercellular adhesion molecule-1 (ICAM-1) and increased endothelial inflammatory markers such as tissue plasminogen activator (tPA) and plasminogen activator inhibitor type 1 (PAI-1). The same authors revealed in a related study that CBC treatment significantly increased the intima-media thickness of the carotid artery and elevated levels of the clotting agent von Willebrand factor in patients undergoing therapy for testicular cancer [60]. Both changes are considered consequences of endothelial damage [61,62]. These effects are compounded by the ability of hydrolysed cisplatin to bind glutathione (GSH), an endogenous antioxidant, thereby reducing the total GSH content and its enzymatic activity. This reduction contributes to elevated ROS levels, which further drive ED [63].

Regarding the hallmarks of ED, emerging research has identified therapy-induced premature ageing as a key contributor to chemo-related cardiovascular complications [64,65]. Among these, senescence, a hallmark of cellular ageing, is now acknowledged as a critical physiological response to cancer treatments.

The development of cellular senescence as a result of medical treatment is termed iatrogenic senescence. Growing in vitro evidence indicates that various chemotherapeutics induce iatrogenic endothelial senescence, and it is now hypothesised that their intravenous route of administration poses a significant risk factor for its onset. Importantly, ageing cells exhibit a senescence-associated secretory phenotype (SASP), characterised by the release of inflammatory markers; cytokines, chemokines, growth factors, and extracellular matrix proteins have all been implicated [66]. In line with this, doxorubicin (DOX) exposure in C57BL/6 J mice has been shown to increase SASP factors such as IL-1β and IL-6 in a manner that correlates with increased aortic stiffness [67]. A recent comparative in vitro study demonstrated that senescent endothelial cells display more pronounced SASP expression compared to epithelial cells and myoblasts, with distinct protein profiles varying across cell types [68]. Consequently, senescent endothelial cells are considered to play a critical role in sustaining chronic inflammation.

Chemotherapy-induced immune-mediated endothelial damage represents an extensive field of research due to the naturally disruptive nature of these medicines in both the malignant and healthy environments. Cisplatin, for example, promotes vascular changes by upregulating the production of pro-inflammatory cytokines IL-1 and IL-6 [69]. IL-1 has long been recognised for its role in activating endothelial cells toward a prothrombotic and pro-inflammatory state [70]. Numerous mouse studies have provided compelling genetic evidence implicating IL-1 as a key driver of atherosclerosis and its complications [71,72,73]. In a similar vein, a recent single-cell sequencing study by Pan and colleagues revealed that cisplatin treatment significantly upregulates inflammatory markers such as *Timp4*, *Tns1*, *Gdf15*, and *Neat1*, while also modulating apoptotic and vessel remodelling genes, including *Fas*, *Bax*, and *PIM-3* [74].

This multitude of evidence underscores the profound impact of chemotherapeutics on endothelial integrity and highlights the pressing need for the development of new therapies to address the cardiovascular challenges faced by cancer survivors.

## 3. What Are Polyphenols?

Polyphenols are naturally occurring antioxidants found in a wide range of sources, including fruits, vegetables, bacteria, and fungi, and are characterised by aromatic rings with hydroxyl groups [75,76]. With over 8000 identified polyphenols, this diverse group of compounds exhibits significant structural variation [77]. Their complexity allows them to remain in crop residues even after industrial processing; thus, these are a promising resource for polyphenol extraction. In Europe alone, substantial quantities of crop waste are produced annually, offering an opportunity for resource recovery and sustainable utilisation [78,79].

These compounds also demonstrate neuro- and cardioprotective effects [80], primarily attributed to their antioxidant [81,82,83] and anti-inflammatory properties [84,85,86]. Additionally, they play crucial roles in modulating cardiomyocyte function, preventing cell apoptosis [87], and shielding against cardiac hypertrophy [88,89], atherosclerosis, and ischemia/reperfusion injuries [81,83,86]. Notably, several polyphenols have shown protective effects against DOX-induced cardiomyopathy, such as quercetin and baicalin, which mitigate DOX-induced cardiotoxicity by reducing oxidative stress and apoptosis while enhancing therapeutic efficacy in cancer treatment [90,91]. Likewise, in vivo studies with isorhamnetin (5 mg/kg), kaempferol (10 mg/kg), and dihydromyricetin (125–500 mg/kg) have demonstrated reduced cardiomyocyte necrosis, ROS production, and lipid peroxidation, while antioxidant enzyme activity has been enhanced [92,93]. 3′,4′-Dihydroxyflavonol (DiOHF) further provides cardiac protection in the case of ischemia/reperfusion injuries by improving myocardial function and coronary blood flow [94].

These studies offer a comprehensive perspective on the effects of polyphenol intake. However, such studies are subject to significant limitations, including small sample sizes, inadequate controls, inconsistencies amongst dietary assessment methodologies, and reliance on heterogeneous databases to estimate polyphenol consumption [95]. To date, several studies have been published claiming overwhelming health benefits with the regular consumption of various food products, but the causative agents have not been sufficiently explored. In the same light, many polyphenols have been described as invoking positive change in a predominantly diseased state, but their clinical use remains modest at best. This gap is partly attributable to the limited knowledge of polyphenol bioavailability and the challenge of identifying the specific compounds responsible for observed biological effects, particularly when multiple polyphenols are present simultaneously [96]. To date, only cocoa and extra virgin olive oil have received approved health claims related to their polyphenol content [96].

### 3.1. Polyphenols: The Underlying Chemistry

To date, over 8000 polyphenols have been described, falling across four broad subgroups: flavonoids, stilbenes, phenolic acids, and lignans [97]. Studies have shown that these molecules can regulate signalling pathways, modify cellular antioxidant status, influence genetic expression, and reshape cellular inflammatory profiles. Here, we propose that polyphenols could be a viable chemotherapy adjunct to mitigate microvascular toxicity.

#### 3.1.1. Phenolic Acids

Phenolic acids are a major class of plant-derived polyphenols generally categorised into two main groups: benzoic acid and cinnamic acid compounds, both commonly sourced from the diet [98]. Compounds derived from hydroxybenzoic acid are characterised by a single carboxylic group attached to a benzene ring. Prominent examples in this category include gallic acid, vanillic acid, and protocatechuic acid. The substituents on the aromatic ring in phenolic acids affect the stabilisation of their structure and consequently influence their radical-quenching ability. In contrast, hydroxycinnamic acid derivatives feature a three-carbon chain attached to the benzene ring framework. These compounds can undergo substitution with hydroxyl groups, enhancing their diversity. Common representatives in this group include p-coumaric acid, ferulic acid, and caffeic acid [99]. These compounds are widely recognised for their antioxidant properties.

The highest concentrations of benzoic acids (measured as the fresh weight) have been recorded in species from the *Apiaceae* family (spices and herbs), with anise containing 730–1080 mg/kg, cumin up to 42 mg/kg, fennel up to 106 mg/kg, and parsley up to 30 mg/kg [100]. In studies involving purified caffeoylquinic acid, effective concentrations contributing to vasoactive efficacy have been reported to range between 138.7 and 900 mg/day [101,102]. However, these findings primarily pertain to purified forms of phenolic acids, as naturally occurring phenolic acids in dietary sources are often not readily bioavailable, especially when consumed in low amounts. For instance, over 99% of phenolic acids in cereals exist in bound forms, linked to arabinoxylan chains through ester bonds or to lignin via ether bonds [103]. Unlike the free hydrolysable phenolic acids in fruits and vegetables—which are mostly degraded or excreted due to their free carboxylic acid group— these bound forms are resistant to hydrolysis by human digestive enzymes and only become bioavailable in the colon, where bacterial enzymes such as microbial esterases release them [104]. Nonetheless, the recovery of phenolic acids in plasma remains lower after cereal intake compared to after the intake of sources such as fruits, coffee, and wine [105]. Nevertheless, phenolic acids possess much higher in vitro antioxidant activity than well-known antioxidant vitamins, like vitamin C or vitamin E [106].

Phenolic acids can undergo biotransformation through processes like the esterification and decarboxylation of the carboxylic group, altering their hydrophilic–lipophilic balance (HLB). This modification increases their ability to cross biological membranes and target active sites in the body, enhancing their medicinal activity [107]. Furthermore, these transformations can be optimised using heterogenous biocatalysts, which may be endogenously produced or co-administered with phenolic acids. These chemical and metabolic traits underscore their potential as bioactive compounds, even when consumed in bound forms with limited initial bioavailability [107]. Furthermore, phase II conjugation processes—including methylation, glucuronidation, and sulfation—modify the phenolic acids’ structure, influencing their biological effects and improving their delivery and bioactivity [108,109]. For example, glucuronidation and sulfation, which target hydroxyl groups, can reduce the antioxidant capacity of phenolic acids, thereby diminishing their therapeutic potential. However, these metabolic modifications also improve compound stability, enhance bioavailability, and enable re-entry into circulation as active metabolites, which is crucial for achieving their desired biological effects.

Phenolic acids demonstrate significantly higher in vitro antioxidant activity compared to well-known antioxidant vitamins due to the reactivity of the phenol moiety, the hydroxyl substituent on the aromatic ring [106]. Beyond their antioxidant properties, numerous epidemiological and experimental studies have highlighted the protective roles of phenolic acids in combating degenerative diseases, including cardiovascular diseases, cancer, diabetes, inflammation, and more. While these compounds are primarily recognised as direct antioxidants, they also exhibit indirect antioxidant activity by inducing endogenous protective enzymes and positively modulating signalling pathways in ED [110], as further detailed in the following section on the mechanism of action of polyphenols.

#### 3.1.2. Flavonoids

**Figure 3:**
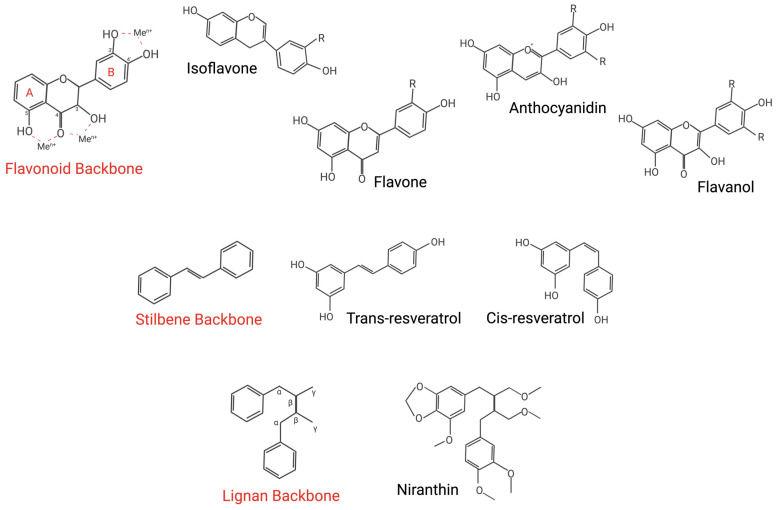
Chemical structures of polyphenols. Many of these compounds require chemical modifications—such as hydrolysis—to exhibit their ‘active’ forms. Structural features, particularly within the flavonoid backbone, enable key functions such as trace metal binding [127,128].

Flavonoids (Figure 3) are polyphenolic compounds composed of two aromatic rings linked by a three-carbon bridge, typically forming an oxygen-containing heterocyclic ring. This structure provides a site for radical incorporation and imparts electrophilic properties, serving as the basis for various flavonoid subtypes with diverse biological activities [111]. Based on their structural characteristics, flavonoids are categorised into six subtypes: anthocyanins, flavones, flavanols, isoflavones, flavanols, and flavanones [112].

These compounds are widespread in over 4000 plant species [113] and exhibit higher bioavailability compared to other polyphenols, which facilitates easier extraction and purification. For instance, flavonoids have been successfully extracted from dandelions, a low-cost and abundant plant source. The general backbone structure of flavonoids, characterised by a C6-C3-C6 arrangement and two phenolic units (C6), contributes to their electroneutral stability, enabling them to form cation–π interactions and limiting the availability of free radicals for hydrolysis. Notably, hesperetin-5′-O-β-rhamnoglucoside, a newly isolated flavonoid, demonstrated remarkable antioxidant activity with an IC50 value of 8.72 mg/L, surpassing the antioxidant potential of many phenolic acids [114]. While it has not yet been utilised clinically, its promising antioxidant properties suggest potential applications in cardiovascular disease, like its analogues within the flavonoid family [115]. This low IC50 value also highlights its increased efficiency and effectiveness at physiologically achievable concentrations, meaning that measurable antioxidant effects can occur in vivo without the need for high doses. This not only enhances its therapeutic potential but also reduces the risk of dose-related toxicity compared to medications like high-dose vitamin C infusions, which are commonly administered to cancer patients. When given as high-dose infusions, vitamin C can lead to unwanted cytotoxic side effects such as kidney damage or oxidative stress [116].

The absorption and bioavailability of flavonoids, such as flavanones, also demonstrate distinct characteristics. For example, the maximum concentration of hesperetin conjugates, such as hesperetin-7-rutinoside, is reached 5–6 h after consumption, consistent with the hydrolysis of the rutinoside moiety in the distal part of the intestine. In one study, the mean maximum plasma concentrations of hesperetin were 0.5 and 1.3 mmol/L for 0.5 and 1.0 L doses of juice, respectively [117]. Similar findings were observed for naringenin, a natural flavonoid, with plasma concentrations reaching 0.4–0.6 mM after the ingestion of grapefruit or grapefruit juice, providing 115 mg of naringenin aglycone equivalent, and 0.5–0.6 mM after the consumption of oranges or orange juice, providing 115 mg of hesperetin aglycone equivalent [118]. Monomeric catechins and flavanones are absorbed efficiently, though they exhibit short half-lives (1.5–7 h). For example, flavanones like hesperetin-7-rutinoside and naringenin-7-rutinoside show a delayed maximum plasma concentration (Tmax), indicating absorption after deglycosylation in the large intestine, which improves bioavailability. In contrast, flavonols tend to have much longer half-lives [119]. This highlights the unique metabolic pathways and bioavailability characteristics of flavonoids, which contribute to their enhanced biological effects, particularly in terms of their antioxidant properties.

These phenolic constituents also inhibit enzymes responsible for superoxide anion production, such as the production of xanthine oxidase and protein kinase C, due to their double benzene ring structure [120,121]. Additionally, many flavonoids can efficiently chelate trace metals, which play an essential role in oxygen metabolism. The proposed binding sites for trace metals in flavonoids include the catechol moiety in ring B, the 3-hydroxyl and 4-oxo groups in the heterocyclic ring, and the 4-oxo and 5-hydroxyl groups between the heterocyclic and A rings. However, the catechol moiety in ring B is considered the primary contributor to metal chelation [122]. Due to their lower redox potentials (0.23 < E7 < 0.75 V), flavonoids are thermodynamically capable of reducing highly oxidising free radicals, such as superoxide, peroxyl, alkoxyl, and hydroxyl radicals, by donating hydrogen atoms [123,124]. This antioxidant capacity is clinically relevant to treating ED, as other available radical scavengers have been associated with side effects such as haemorrhagic stroke (vitamin E), nosebleeds (vitamin E; low-quality evidence), skin rashes (multivitamins; moderate-quality evidence), and the potential promotion of lung carcinogenesis (vitamin C in women, vitamin A in smokers, and β-carotene in those exposed to asbestos; high-quality evidence) [125,126]. Additionally, ACE inhibitors and ARBs, while effective in reducing shear stress in the vasculature, can lead to hyperkalaemia and nephrotoxic side effects.

Nevertheless, while flavonoids demonstrate significant promise as cardioprotective agents, translating these benefits into consistent clinical outcomes remains a challenge. Their clinical impact is limited by difficulties in isolating specific subtypes, inconsistent effects on vascular parameters, and inconclusive evidence regarding the biological activity of their metabolites [129]. Previous research has linked flavanone metabolites to endothelial health, but further investigations are needed to validate and elucidate these effects.

#### 3.1.3. Stilbenes

Stilbenes are a class of phenylpropanoids produced by various plant species, including peanuts, grapevines, and sorghum, among others [130]. Their structure is illustrated in Figure 3. These compounds are typically present in low levels in the human diet, as they primarily function as antimicrobial phytoalexins in plants, produced in response to injury or infection [131].

Stilbenes exhibit diverse applications due to their ability to form polymers and copolymers using their hydroxyl radicals. This property makes them promising agents for reducing ROS. Their antioxidant potential is derived from their unique structural composition, including an aromatic ring, conjugated double bonds, and several functional groups that contribute to their reactivity and ability to scavenge hydroxyl reactive groups [132].

Resveratrol (3,5,4′-trihydroxy trans-stilbene) is one of the most extensively studied stilbenes. Predominantly found in grapes and grape-derived products, resveratrol has garnered significant attention from the pharmaceutical, food, and cosmetic industries due to its broad range of health benefits [133,134]. However, its concentration in foods is highly variable, influenced by factors such as grape cultivars [135]. Nevertheless, resveratrol has been synthesised in a four-step synthesis process with a 93% yield using commercially available 3,5-dimethoxy-1-ethynyl-benzene and 4-iodoanisole, followed by reduction, isomerization, and deprotection and with an overall yield of 63% [136]. This efficient method demonstrates significant potential for large-scale production at reduced costs. Additionally, biotechnological approaches using various host systems are emerging as promising alternatives for sustainable and cost-effective production, making this the most attractive agent in the stilbene family [137].

Conversely, the use of resveratrol and other stilbenes as oral therapeutic agents is significantly limited by their poor aqueous solubility (<0.05 mg/mL for resveratrol) and low bioavailability, primarily due to rapid metabolism by phase II enzymes [138]. Resveratrol’s structure, which includes two aromatic rings and a resveratrol backbone, lacks sufficient polar functional groups to form hydrogen bonds, impairing its solubility. This challenge is not unique to resveratrol; stilbenes like pinosylvin, gnetol, and isorhapontigenin share structural features, such as a high density of hydroxyl groups on their aromatic rings. These features make their molecular structure susceptible to an electrophilic attack, leading to degradation and further complicating solubility, absorption, and distribution in human subjects [139].

Still, resveratrol’s high fat solubility allows it to effectively permeate the cell membrane. Studies have shown that approximately 75% of an oral dose of C14-labelled resveratrol is absorbed. Consequently, the hepatic uptake with in vitro models using the HepG2 hepatoblastoma cell line demonstrated a maximum efficiency of 80%, showcasing the liver’s role as the primary site of accumulation for this polyphenol and highlighting its ability to bypass primary metabolism [140,141]. However, such detailed pharmacokinetic studies are limited to resveratrol, leaving significant gaps in our understanding of the absorption and distribution of other stilbenes. Resveratrol and structurally similar stilbenes such as oxyresveratrol and astringin can undergo extensive biotransformation. For instance, when a mixture of resveratrol derivatives (R3S and R4S in a 3:2 ratio) was administered to mice, it resulted in significant biotransformation, producing glucuronides, disulfates, and resveratrol itself across various tissues, including the plasma, intestinal mucosa, liver, lungs, and pancreas. In plasma, the peak levels of resveratrol were found to be approximately 20% of the maximum concentration observed when measured alongside a mixture of modified resveratrol monosulfates [142]. Resveratrol, however, has a significant anti-inflammatory capacity. Although direct comparisons in the context of cardiovascular dysfunction have not yet been made, studies in COPD patients showed that resveratrol more efficiently reduced the release of inflammatory mediators compared to dexamethasone, a commonly used corticosteroid, in human airway smooth muscle cells (HASMCs) [143].

Despite these challenges, the extensive research on resveratrol offers valuable insights into optimising the therapeutic potential of stilbenes. Advancing synthetic and biotechnological production methods, alongside developing innovative delivery strategies, could significantly enhance the clinical utility of this promising class of compounds.

#### 3.1.4. Lignans

Lignans are a class of plant-derived polyphenols distinct from lignin, which is a structural polymer integral to plant cells. Unlike lignin, lignans are bioactive, non-caloric, and synthetic resin compounds. Structurally, they consist of a 2,3-dibenzylbutane backbone formed by the dimerisation of two cinnamic acid residues, derived from coniferyl alcohol precursors. These biphenolic compounds exhibit an oestrogen-like structure, classifying them as phytoestrogens with notable antioxidant properties [144,145]. Henceforth, due to chemical composition, these compounds can act as selective oestrogen receptor modulators (SERMs). As a result, they are believed to exert anti-cancer effects, particularly against hormone-related cancers [146]. They could potentially compete with emerging therapies like JAK-STAT inhibitors and microRNA-based therapies, as they are more cost-effective to produce and also capable of inducing transcriptomic changes. Examples include secoisolariciresinol diglucoside (SDG), secoisolariciresinol (SECO), pinoresinol, matairesinol, and others [147,148].

Linseed (flaxseed) is an exceptionally rich source of SDG, a glycosylated form of SECO, containing up to 800 times the SDG concentration of other foods. Its high lignan content and the scalability of flaxseed cultivation make it a valuable resource for therapeutic and industrial applications. Flaxseed is cultivated in over 50 countries, with major producers including Kazakhstan, Russia, and Canada, while India contributed approximately 121 million tonnes to global production in 2020 [149,150]. Additionally, a synthetic lignan, LGM2605, derived from widely available vanillin, offers a scalable and practical alternative [151].

SDG, often referred to interchangeably with SECO, exists in mono- (SMG) or di-glycosylated forms. These molecules are interlinked with hydroxy-methylglutaryl (HMG) units, forming a biopolymer. This polymer can be broken down by gut microbiota into active mammalian lignans like enterodiol (ED) and enterolactone (EL), significantly enhancing their bioavailability and pharmacological efficacy. The extraction of SDG from defatted linseed meal has a yield of approximately 3%, underscoring its potential as a pharmacologically active agent [152]. This property reduces degradation in the ileal epithelium, allowing hydrolysis to release the monomer subunit, which can then interact with specific targets effectively. Henceforth, from these two points, we can see how lignan production is already feasible for worldwide use, and its polymeric nature provides an advantageous route for improving bioavailability.

## 4. Polyphenols’ Therapeutic Mechanisms

Chemotherapeutic agents used to treat cancer can exert significant stress on the vascular endothelium, often resulting in pathological changes that contribute to cardiovascular disease. These disruptions are primarily linked to elevated levels of ROS, impaired NOS signalling, the altered gene expression of key homeostatic regulators such as VEGF, cellular senescence, heightened inflammatory responses, and modifications to the ECM of the vasculature. Polyphenols have shown potential in mitigating adverse effects by targeting these processes, offering a promising avenue for reducing chemotherapy-induced vascular damage.

### 4.1. Modulation of Oxidative Stress and eNOS

ROS play a dual role in cellular function, serving as both critical mediators of signalling pathways and potential instigators of oxidative stress [153]. In the context of chemotherapy, the cytotoxic effects of these agents not only target cancer cells but also inadvertently affect healthy tissues, leading to a substantial increase in ROS production. This systemic oxidative stress particularly impacts the vascular endothelium, which is highly susceptible to ROS-induced damage due to its relatively limited antioxidant defence reserves compared with other body tissues [154].

NO, though a member of the ROS family, plays a crucial role in vascular smooth muscle cells (VSMCs) and is produced via the eNOS pathway. In a healthy endothelium, the NO availability and eNOS activity are key indicators of vascular health, as they promote endothelial function and facilitate vasodilation. However, under oxidative stress, the NO availability diminishes due to its reaction with superoxide anions, forming peroxynitrite. This reduction in NO levels impairs endothelium-dependent relaxation, leading to decreased vascular function [155].

Polyphenols primarily act as antioxidant agents and ROS scavengers, a capability rooted in their unique molecular structure, as mentioned at the beginning of the previous section. Stilbenes, characterised by their phenol groups, can exist in a free form or as prenylated, geranylated, or glycosylated derivatives. These molecules can also form multimeric chains, polymers, and copolymers [156], resulting in a high density of aromatic compounds within a structurally reduced framework. The phenolic groups in stilbenes are particularly effective at donating hydrogen atoms to neutralise ROS or transferring electrons to lower the activation energy required for radical reactions. For instance, the π-electrons in the aromatic rings of resveratrol have been identified as key antioxidant targets [157,158], facilitating the neutralisation of free radicals. Interestingly, the addition of an extra hydroxyl group to the phenolic moieties can greatly enhance their antioxidant potential. Astringinin, a resveratrol analogue with an additional hydroxyl group, exhibited a remarkable 160-fold increase in its scavenging capacity for the superoxide anion compared to resveratrol, as demonstrated in a study by Hung et al. This superior activity was further validated in experiments involving Wistar-Kyoto rat hearts subjected to ischemia–reperfusion injury. Pre-treatment with an astringinin infusion (10 µM) significantly reduced the infarct size and improved coronary flow recovery compared to resveratrol [159]. Furthermore, the central double bond in stilbenes promotes π–π interactions, which plays a crucial role in stabilising free radicals [160]. These unique structural features enable stilbenes to effectively counter oxidative stress in cellular environments.

Interestingly, the structure of resveratrol allows it to bind to the active site pocket of the PI3K receptor on the cell surface, triggering the downstream activation of Akt. Akt subsequently phosphorylates eNOS, enhancing NO-dependent vasodilation and improving endothelial function [161,162,163]. Building on these mechanisms, Bhatt et al. investigated the effects of resveratrol on eNOS expression and coupling in Wistar-Kyoto (WKY) rats and Spontaneous Hypertensive Rats (SHRs). In their study, young (3–4 weeks) SHRs and WKY rats were administered resveratrol in drinking water for 10 weeks, with untreated rats serving as controls. By the end of the treatment period, control SHRs exhibited elevated blood pressure, oxidative stress, and impaired endothelium-dependent relaxation compared to WKY rats. Using fluorescence spectroscopy and a nitrite/nitrate assay, the study demonstrated that ED in SHRs was associated with reduced nitrite/nitrate levels and eNOS uncoupling, hallmarks of disrupted NO signalling. However, resveratrol treatment effectively reversed these abnormalities. It restored endothelial-dependent vasorelaxation, normalised nitrite/nitrate levels, and promoted proper eNOS coupling, significantly improving endothelial function in both SHRs and WKY rats compared to their untreated counterparts [164]. Their results revealed that resveratrol treatment significantly coupled eNOS expression, restoring NO bioavailability in both SHRs and WKY rats compared to untreated controls.

**Figure 4:**
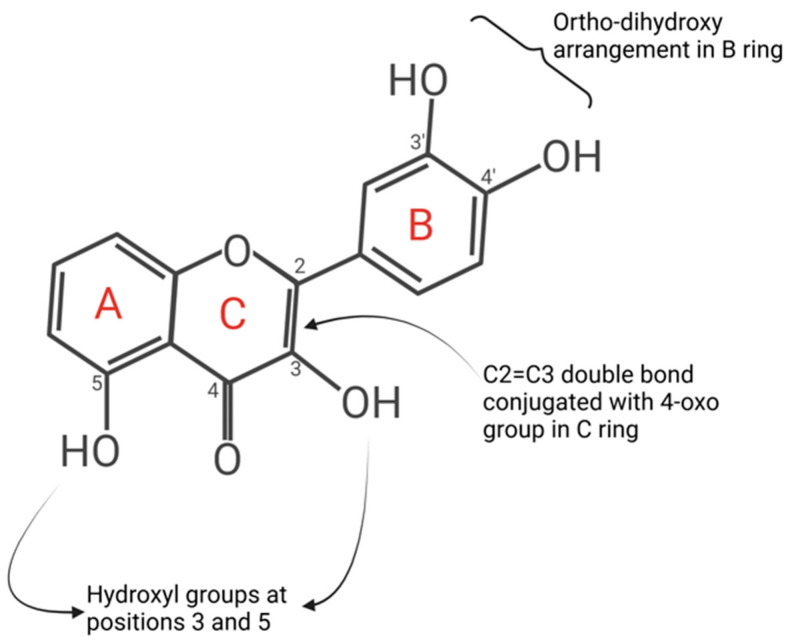
Structural features of flavonoids that confer antioxidant ability.

Comparatively, flavonoid compounds demonstrate significant antioxidant activity through the elevation of uric acid concentrations, metal chelation, and the enhancement of low-molecular-weight antioxidant activity (Figure 4) [165]. Their radical scavenging efficiency stems from specific structural features: an ortho-dihydroxy arrangement in the B ring (enabling electron delocalisation), a 2,3-double bond conjugated with a 4-oxo group in the C ring (facilitating electron delocalisation across the molecule), and hydroxyl groups at positions 3 and 5 (supporting hydrogen bonding with the oxo group) [166]. Furthermore, the antioxidant capacity of flavonoids can be amplified in vitro through polymerisation, as seen with proanthocyanidins—polymers of catechins—known for their high hydroxyl group density [167]. Notably, flavonoids such as quercetin and baicalin have demonstrated potential in mitigating DOX-induced cardiotoxicity by reducing oxidative stress and apoptosis while simultaneously enhancing the therapeutic efficacy of cancer treatments [111,168]. In a randomised, controlled, single-blind, crossover trial, Lyall et al. reported that flavonoid-rich blood orange juice (BOJ) significantly improved FMD, indicating favourable effects on endothelial function in overweight healthy participants. The study included 15 participants (mean BMI: 28.3 ± 3.1 kg/m^2^), who consumed either BOJ or a sugar-matched control drink (CD) (200 mL twice daily) for 2 weeks, with a 1-week washout period between treatments. The FMD was assessed before and after each intervention following an overnight fast. The findings revealed a significant improvement in FMD in the BOJ-treated group compared to the CD group, underscoring the vascular benefits of BOJ. These effects were attributed to the synergistic actions of anthocyanin and flavanone metabolites highly concentrated in the BOJ beverage, which enhanced NO bioavailability through the modulation of endothelial pathways and a reduction in oxidative stress [169]. However, it is important to note that the study’s small sample size may limit the generalizability of the results, even though confounding factors were addressed and secondary markers of ED were evaluated.

Conversely, in other randomised clinical trials, the acute consumption of high-flavanol cocoa powders (918 mg) incorporated into a fatty meal was shown to mitigate the impairment of FMD caused by the meal, compared to the consumption of a low-flavanol cocoa powder (28 mg) in healthy individuals (n = 18). While the precise mechanism by which flavanols improve ED remains unclear, the effect did not appear to be related to changes in triglyceride or free fatty acid levels [170]. Similarly, another randomised trial demonstrated that ice cream enriched with cocoa powder, hazelnuts, and green tea (providing a high polyphenol content of 1817 mg) acutely reduced circulating markers of oxidative stress (serum hydroperoxides and H_2_O_2_) and significantly improved both FMD and the reactive hyperaemia index (RHI) in healthy participants (n = 14) [171].

Building on this, Li et al. hypothesised that naringenin could ameliorate endothelial injury through a mitochondria-dependent pathway. In human umbilical vein endothelial cells (HUVECs)—a widely used model for studying endothelial function—naringenin was shown to inhibit the generation of ROS in both mitochondria and the cytoplasm following homocysteine-induced endothelial injury. Additionally, it restored the mitochondrial membrane potential by activating the AMPKα/Sirt1 signalling pathway [172]. SIRT1, a NAD-dependent deacetylase, requires NAD as a cofactor to catalyse deacetylation reactions, which regulate cellular stress responses. Under oxidative stress, NAD levels are depleted, impairing SIRT1 activity; however, naringenin acts as an electron donor, helping to sustain the enzyme’s function. AMPKα activation enhances Sirt1 activity by increasing intracellular NAD+ levels, as it stimulates ATP production in mitochondria [173]. In vivo, endothelial injury—characterised by plasma homocysteine levels exceeding 15 μmol/L—was significantly reduced with naringenin treatment. Naringenin effectively minimised arterial wall damage and preserved endothelial function, with its mechanism in vivo aligning with the findings observed in vitro [173].

Polyphenols not only function as direct antioxidants but, as demonstrated in previous examples, also influence key cellular pathways. These include the modulation of transcription factors, gene expression, and enzymes that regulate ROS levels in the cytoplasm, support mitochondrial health in the endothelium, and enhance eNOS function. For instance, treatments with quercetin or its derivative, 3′-O-methyl quercetin (30 µM), as well as rhamnetin (1, 3, 5 µM), derived from the berries of Rhamnus petiolaris, have been shown to reduce ROS production and decrease cell death in rat and H9c2 cardiomyocytes exposed to hydrogen peroxide (H_2_O_2_). This protective effect is linked to the upregulation of the activity of several key antioxidant enzymes, including NAD(P)H:quinone oxidoreductase 1 (NQO-1), heme oxygenase 1 (HO-1), thioredoxin reductase 1 (TR), and total glutathione S-transferase (GST) activities [174]. Moreover, polyphenols show a promising ability to enhance endothelium-mediated dilation by interacting with numerous upstream kinases that phosphorylate eNOS at stimulatory sites. Kinases identified in in vitro studies include the PI3K/Akt axis, MAPK, AMPK, and CAMKII [175,176]. Additionally, polyphenols like quercetin and rhamnetin mitigate oxidative stress by inhibiting the activation of MAPKs, which are involved in inflammatory responses and cellular stress pathways [174,177]. Similarly, in an in vivo two-kidney, one-clip (2K-1C) rodent model of hypertension, Boonla et al. demonstrated that curcumin reduces NOX2 expression and increases eNOS protein levels, with the resulting rise in eNOS expression contributing to the restoration of endothelial hemodynamic function [178].

Lignans, due to their lipophilic nature and structural similarity to oestrogens, can cross the plasma membrane and activate the master regulator Nrf2, facilitating its translocation to the nucleus [179]. Nrf2, a transcription factor highly sensitive to oxidative stress, plays a crucial role in promoting the transcription of numerous antioxidant genes. Under normal conditions, Nrf2 is sequestered in the cytoplasm by Keap1 through its cysteine residues [179]. Lignan polyphenols and chemopreventive flavonoids are known to interact with Keap1 by adding chalcones to its thiol groups via a Michael-type reaction. This interaction releases Nrf2, allowing for its translocation to the nucleus, where it upregulates the expression of key antioxidant enzymes such as SOD, CAT, and NQO1 [180].

Similarly, stilbenes can also regulate the gene expression of protective enzymes through an electrophile-responsive element (EpRE), inducing Nrf2 expression in cells [181]. Resveratrol, a compound from the stilbene family, interacts with multiple molecular targets to exert potent antioxidant and anti-inflammatory effects [182]. For instance, resveratrol protects against H_2_O_2_-induced oxidative stress by activating the SIRT1-dependent autophagy pathway in endothelial cells. In endothelial cell models, resveratrol was shown to upregulate SIRT1 levels in a concentration-dependent manner. Consequently, when the SIRT1 gene was knocked out, its antioxidative and anti-apoptotic effects were diminished [183]. Supporting this, a randomised, double-blind, placebo-controlled crossover study investigated the effects of resveratrol (400 mg/day) on endothelial function in twenty-eight adults with stage 3 chronic kidney disease (CKD) and diabetes. Endothelial function, assessed through the brachial artery flow-mediated dilation, significantly improved with resveratrol supplementation, demonstrating its ability to enhance vascular health and reduce cardiovascular risk [184]. A notable limitation, however, is the relatively small sample size, consisting of only 28 individuals.

SIRT1 has also been known to increase the activity of FOXO transcription factors which upregulate eNOS expression [185]. In vitro experiments on EA.hy 926 cells showed that resveratrol treatment enhanced the expression of FOXO transcription factors, resulting in the increased expression of eNOS [186]. Furthermore, the overexpression of SIRT1 within the endothelium has been shown to cause enhanced eNOS expression in apolipoprotein E-deficient mice, leading to a decrease in atherosclerosis [187]. It is thought that SIRT1 deacetylates eNOS, thereby enhancing its activity [188].

Furthermore, a randomised, double-blind trial evaluating the effects of a daily dose of 250 mg clovinol, a stilbene-based polyphenol mixture, versus synthetic glutathione (250 mg/day for 84 days) in healthy adults (n = 70) with metabolic syndrome over a 12-week period showed that clovinol significantly enhanced SOD, GPx, and GSH protein levels in plasma. These changes were correlated with increased Nrf2 activity, as measured via ELISA assays of serum samples, alongside a higher GSSG/GSH ratio, an indicator of improved intracellular redox homeostasis [189]. These findings suggest that polyphenol mixtures like clovinol possess the capability to modulate cellular transcriptomes, inducing phenotypic changes that promote oxidative stress resilience and metabolic regulation, rather than merely acting as direct antioxidants. However, the study presents some limitations that merit consideration. Notably, the intervention period was relatively short—30 days—which precludes conclusions about the long-term effects and safety of clovinol. Additionally, the trial did not account for participants’ dietary habits, physical activity, or other lifestyle factors in the inclusion criteria, which could have influenced the outcomes.

### 4.2. Controlling the Peptide Environment

Polyphenols exert many of their biological actions by influencing transcriptional networks and signalling cascades that regulate gene expression. These mechanisms enhance the production of anti-inflammatory mediators and NO, counteracting the adhesive interactions between vascular and immune cells. As a result, they help mitigate or prevent ED.

Polyphenols exert protective effects on the endothelium by modulating immune cell recruitment and adhesion molecule expression, thereby reducing inflammation and vascular damage. One key mechanism involves limiting the homing of inflammatory cells from circulation and downregulating endothelial adhesion molecules, such as E-selectin and ICAM-1 [190,191]. This suppression prevents excessive immune–endothelial interactions and reduces cellular migration into the subendothelial space, a process linked to atherosclerotic plaque formation [192,193].

Various in vitro studies have further supported this role, demonstrating that polyphenols attenuate ED by reducing adhesion molecule overexpression. Notably, Zhang et al. found that the stilbene resveratrol significantly reduced tumour necrosis factor-α (TNF-α)-induced NF-κB activation and IκB-α phosphorylation in bone marrow-derived endothelial progenitor cells [194]. This inhibition led to a dose-dependent decrease in VCAM-1, ICAM-1, and E-selectin expression, suggesting that polyphenols can disrupt inflammatory signalling pathways that contribute to ED. Treatment with resveratrol decreased VCAM-1, ICAM-1 and E-selectin in a dose-dependent manner.

Endothelin-1 (ET-1) is a potent vasoconstrictor molecule released from endothelial cells [195,196], and its overproduction is associated with hypertension, atherosclerosis, and ischemic heart disease [195,197]. While NO and natriuretic peptides help regulate ET-1 release, its dysregulation contributes to inflammatory cytokine production, including of TNF-α, IL-1, and IL-6, as well as increased ROS formation [198,199]. Importantly, a recent review of 54 meta-analyses and randomised controlled trials revealed that the intake of the diarylheptanoid polyphenol, curcumin, significantly lowered IL-6 levels in five of eight studies and reduced TNF-α levels in six of nine studies [200], further highlighting its role in mitigating ET-1-driven inflammation.

Emerging evidence suggests that polyphenols may directly suppress ET-1 expression, offering potential therapeutic benefits for ED. Liu et al. demonstrated that resveratrol treatment (1–100 μM) in endothelial cells reduced strain-induced ET-1 secretion, ET-1 mRNA expression, and ET-1 promoter activity [201]. This effect was partially mediated via ERK1/2 pathway inhibition, likely through a reduction in ROS formation. Similarly, Storniolo et al. reported that hydroxytyrosol and polyphenol-rich extracts from extra virgin olive oil reversed ET-1 overproduction in endothelial cells exposed to high levels of glucose and fatty acids [202]. However, the specific polyphenols responsible for this effect were not identified. Further supporting these findings, Khan et al. demonstrated that red wine polyphenols exerted a concentration-dependent inhibition of ET-1 synthesis in cultured bovine aortic endothelial cells, with resveratrol (30 μM) and piceatannol (30 μM) significantly suppressing ET-1 production [203]. Thus, the attenuation of ET-1 activity using polyphenols may serve as a protective method for counteracting chemotherapy-induced ED.

Beyond ET-1 modulation, angiotensin II represents another critical factor contributing to ED [204]. While ET-1 and angiotensin II share overlapping pathways in promoting vascular inflammation and oxidative stress, angiotensin II exerts distinct detrimental effects on the vascular wall, primarily through its interaction with the angiotensin II type 1 (AT1) receptor. In addition to inducing vasoconstriction and elevating blood pressure, angiotensin II promotes the generation of ROS, leading to NO degradation and ED [205]. Furthermore, oxidative stress triggered by angiotensin II reduces eNOS activity, particularly through monocyte-dependent S-glutathionylation, impairing NO bioavailability [206]. This oxidative imbalance can further amplify angiotensin II levels, creating a self-perpetuating cycle of vascular dysfunction. For instance, a study using male Sprague Dawley rats treated with Nω-nitro-L-arginine methyl ester (L-NAME), an NO inhibitor, found that these rats developed increased blood pressure, elevated angiotensin II levels, heightened oxidative stress, and elevated IL-6 levels [207]. This interplay highlights how circulating angiotensin-converting enzyme (ACE) within the endothelium contributes to a self-perpetuating cycle, where oxidative stress—exacerbated by chemotherapeutic agents—enhances vascular dysfunction. Notably, polyphenols have been shown to interact with ACE, potentially mitigating these detrimental effects.

Polyphenols can act as inhibitors of the ACE active site. In a recent study, a mixture of isolated flavonoids was evaluated for its inhibitory potential. Several compounds demonstrated ACE inhibition with IC50 values ranging from 7.3 to 43.8 µM, using quercetin (IC50 = 25.2 ± 0.2 µM) as a positive control. Their inhibition kinetics were identified as noncompetitive [208]. Notably, isoorientin emerged as a particularly potent ACE inhibitor (IC50 = 7.3 ± 0.1 µM, Ki = 6.6 ± 0.1 µM). Structural analysis revealed that free hydroxyl groups on flavone moieties and glucose linkages in the A-ring were key determinants of ACE inhibition. Additionally, resveratrol has been shown to modulate the renin–angiotensin system. In a study using C57BL/6 mice—a widely used model for studying cardiovascular function—vascular smooth muscle cell (VSMC) biopsies and thoracic aorta samples were collected after six months of treatment with resveratrol. Resveratrol administration led to a reduction in serum Ang II levels and downregulated aortic ACE expression [209]. These findings suggest that resveratrol has the capacity to modulate gene expression related to ACE activity, contributing to vascular protection. Polyphenols show promise as natural regulators of ACE activity. Their ability to mitigate Ang II-mediated ED suggests a potential therapeutic avenue for vascular health.

### 4.3. Attenuating Cellular Senescence

As outlined previously, endothelial senescence increases as a consequence of chemotherapeutic intervention. Polyphenols may help mitigate these effects, potentially preserving endothelial integrity and reducing vascular inflammation.

Flavonoids have been recognised as potent agents against cellular ageing, even in clinical settings. Notably, phase I clinical trials in patients with diabetic kidney disease (DKD) [210] and idiopathic pulmonary fibrosis (IPF) [211] demonstrated that the combination of dasatinib and quercetin (D+Q) reduces the expression of p16^INK4A^ and senescence-associated β-galactosidase (SA-β-gal), hallmarks of senescence that signify irreversible cell cycle arrest and ageing-related dysfunction. In DKD patients (n = 9), a three-day D+Q treatment (100 mg/day dasatinib, 1000 mg/day quercetin) led to a measurable reduction in senescent cells within 11 days. This was accompanied by a decline in SASP factors, which drive chronic inflammation and tissue deterioration. Additionally, skin biopsies also showed a decrease in p16^INK4A+^ and p21^CIP1+^ cells, indicating a reduction in cells that have permanently exited the cell cycle [210]. Similarly, in IPF (n = 14) patients, intermittent D+Q therapy (100 mg/day dasatinib, 1250 mg/day quercetin, three days per week for three weeks) correlated with functional improvements and a reduction in SASP-related inflammatory markers, with 23 out of 48 measured markers showing a correlation coefficient of r ≥ 0.50 [211].

In a recent in vivo study by Zumerle et al., the authors explored the effects of a standardised extract containing dietary flavonoids, specifically luteolin and luteolin-7-O-glucuronide, on the lifespan of naturally aged mice [212]. These mice, starting at 20 months old, were treated with the extract in their drinking water, while the control group received water only. The flavonoid extract significantly reduced markers of ageing in mouse models, including muscle strength and fur thickness, and led to an extension in lifespan. It also decreased the expression of senescence-associated genes and proteins, such as p16^INK4a^, and their interaction with CDK6—an effect evaluated to assess the impact of phenolic compounds on cell cycle regulation—compared to untreated controls. Additionally, using a p16LUC senescence reporter mouse model, they administered doxorubicin, which induced senescence as indicated by increased luminescence from the marker gene after seven days. However, pre-treatment with the flavonoid extract (0.5 mg/kg via oral gavage) starting three days before chemotherapy administration notably reduced the accumulation of p16-positive senescent cells and prevented body weight loss [212]. The precise mechanisms behind the anti-ageing effects of flavonoids remain unclear, and more research is needed to validate their clinical applications in humans.

In contrast, in vitro studies focusing on endothelial senescence have provided further insights into how polyphenols can mitigate or delay the process. For example, a polyphenol-rich Crataegus extract has been shown to effectively delay replicative endothelial senescence by preventing the downregulation of eNOS production. This effect is associated with the reduced expression of key cell cycle regulatory proteins (p53, p21, and p16), pro-oxidant enzymes (NADPH oxidase, COX-1, and COX-2), and components of the local angiotensin system [213]. Moreover, paeonol, a phenolic acid, protects endothelial cells from oxidative stress-induced senescence, while selaginellin counters homocysteine-induced senescence, partly through their antioxidant effects and increased expression of Sirt1 [214]. Resveratrol has also been found to prevent high-glucose-induced endothelial senescence by reducing the generation of ROS and activating the AMPK/Sirt1 and p300/p53/p21 signalling pathways [215]. Interestingly, many of the pathways activated by polyphenols, such as antioxidant defence and eNOS modulation, overlap with those discussed earlier in the review, suggesting a complex interplay between these systems. This cross-relation indicates that polyphenols may not only reduce oxidative stress but also enhance NO production and activate pathways that promote endothelial cell survival and repair.

## 5. Future Perspectives

Throughout this review, we have looked to outline the significant advantages displayed by polyphenols used as therapeutics for both the prevention and treatment of ED following chemotherapy. Despite these advantages, polyphenols remain underused within the clinical setting. The reasons for this are partially attributable to the known limitations that these compounds exhibit, such as poor bioavailability and absorption [129,216]. Despite this, we argue that further research should be conducted to consider the use of polyphenols in early and late cancer care.

Flavonoids, particularly flavanones like hesperetin and naringenin, exhibit distinct pharmacokinetic properties that enhance their clinical relevance. For instance, hesperetin-7-rutinoside undergoes delayed absorption in the large intestine following deglycosylation, resulting in a time to Tmax of approximately 5–6 h [217,218]. Despite their relatively short elimination half-lives (1.5–7 h), flavanones can still achieve plasma concentrations within a biologically active range, as observed with hesperetin and naringenin following orange or grapefruit juice consumption [217,218]. In contrast, flavonols like quercetin exhibit longer half-lives of up to 20 h, potentially offering sustained therapeutic effects with less frequent dosing [219].

Other challenges include chemical instability. The conjugated double bond within the structure of stilbenes, for example, predisposes them to isomerisation between trans- and cis-forms, with the cis-isomers typically exhibiting reduced pharmacological potency [220]. This adds another layer of complexity, as the trans-isomer is generally considered more stable and effective in exerting therapeutic effects. These combined factors—low aqueous solubility, chemical instability, isomerisation, and rapid metabolism—pose significant obstacles to the clinical use of stilbenes like resveratrol. Nevertheless, once absorbed, their hydrophobicity facilitates binding to plasma proteins, enabling transport to their sites of action while simultaneously complicating their excretion.

### 5.1. Overcoming Bioavailability Concerns

Despite promising therapeutic properties, polyphenols are significantly limited by poor water solubility, chemical instability, and extensive first-pass metabolism, which severely restrict their bioavailability and clinical utility. Addressing these challenges has become a central focus in translational research, with considerable effort devoted to the development of advanced drug delivery systems. Among these, nanoparticle-based formulations represent the most extensively studied strategy and have demonstrated promise in enhancing systemic exposure, protecting compounds from degradation, and improving target tissue penetration [221,222].

Nanoparticles function as theranostic platforms capable of circumventing early metabolic clearance and renal filtration, thereby prolonging the circulation time and enabling controlled release. For instance, the nanoencapsulation of quercetin using poly-D,L-lactide has been shown to improve water solubility while maintaining antioxidant properties comparable to those of the free compound [223,224]. Similarly, micellar co-delivery systems containing curcumin and resveratrol have reduced ROS production and apoptosis in cardiomyocytes subjected to doxorubicin-induced toxicity [225]. These findings underscore the versatility of nanoparticle systems in improving both the pharmacokinetic and therapeutic profiles of polyphenolic compounds.

In the context of anthocyanin delivery, tannic acid and poloxamer 188 core–shell particles (TA-F68) have demonstrated a 15.5% increase in retention rates compared to conventional delivery approaches. The release of anthocyanins from these nanoparticles has been found to be esterase-sensitive, offering a mechanism for site-specific drug liberation in enzymatically active tissues [222]. These protective features are critical in mitigating gastrointestinal degradation, which remains a key barrier to oral polyphenol efficacy.

Beyond polymeric and micellar systems, metal–polyphenol nanoparticles have also garnered attention due to their multifunctional capabilities. Zhou et al. recently synthesised nanoparticles using epigallocatechin gallate (EGCG)—a well-characterised flavonoid from the catechin family—by chelating it with CuCl_2_ and subsequently modifying the complex with membrane-penetrating peptides. This dual-functional platform enabled improved lysosomal escape and mitochondrial targeting without compromising biocompatibility [226]. The ability to fine-tune subcellular distribution is of particular interest in targeting mitochondrial dysfunction in cancer and degenerative diseases.

Liposome-based carriers provide an alternative strategy to these conventional polymeric and micellar delivery systems. Characterised by their ability to encapsulate both hydrophilic and lipophilic drugs, these phospholipid bilayer vesicles enhance intestinal absorption and bioavailability while protecting labile molecules from degradation [227]. Phytosomes, which combine polyphenols with phospholipids, and niosomes, formed from non-ionic surfactants, have both been investigated for their ability to improve pharmacokinetic profiles and provide sustained drug release [228]. These formulations enhance the stability of polyphenols and promote more efficient absorption across biological membranes, thereby increasing the systemic availability [228]. Niosomes are particularly attractive due to their greater chemical stability, lower cost, and improved ease of functionalisation compared to liposomes [229]. Likewise, hydrogels—especially those incorporating natural polymers like chitosan or alginate—offer a promising platform for the localised, controlled release of polyphenols, particularly in wound healing and tissue regeneration contexts [230,231].

In parallel, biopolymeric nanoparticles—particularly those derived from natural proteins and polysaccharides—have emerged as promising carriers for compounds like EGCG, offering a greater loading capacity and reduced toxicity compared to synthetic polymer-based systems [232]. Thus, they are an attractive option for improving polyphenol delivery.

Complementary to physical encapsulation technologies are molecular enhancement strategies, which focus on inhibiting metabolic degradation. Piperine, a widely studied bioenhancer, improves the absorption of curcumin and EGCG by suppressing P-glycoprotein and cytochrome P450 enzymes, key regulators of intestinal and hepatic metabolism [233]. In human trials, non-caloric sweeteners such as stevia and sucralose have also demonstrated favourable effects on polyphenol absorption when compared to sucrose, possibly by modulating gastrointestinal transport mechanisms [234]. Such strategies not only enhance the systemic availability but also complement emerging chemical modifications aimed at improving pharmacokinetics and tissue-specific delivery.

Chemical modifications further expand the potential of polyphenol-based therapies. Acylation, glycosylation, and methylation have been shown to enhance lipophilicity and metabolic stability. For example, quercetin acylated with oleic acid via lipase-catalysed enzymatic reactions resulted in a 152% increase in hydrophobicity and significant improvements in drug delivery efficiency [235]. These modifications may play a critical role in tailoring pharmacodynamics and tissue selectivity in future formulations.

Taken together, these innovations represent a paradigm shift in polyphenol pharmacology, moving from isolated compounds to engineered therapeutic systems. Hybrid strategies, such as combining niosomes with hydrogels for localised delivery, demonstrate that synergistic platforms may further optimise drug distribution, retention, and release profiles [225,228]. However, despite promising preclinical data, clinical trials and large-scale manufacturing remain critical bottlenecks. Bridging this gap will be essential to realising the full therapeutic potential of polyphenols in human disease.

### 5.2. Looking Beyond Chemotherapy

In this review, we have discussed the anti-inflammatory properties of polyphenols and their role in modulating the expression of cytokines and adhesion molecules. In 2011, Hanahan and Weinberg updated their pivotal paper, the “Hallmarks of Cancer”, to include new ‘emerging’ hallmarks [236,237]. Included within these was the ability of the inflammatory environment to promote tumorigenesis, leading to large bodies of research looking to eradicate such conditions in the clinical setting. Advances in cancer care over the past two decades have seen the introduction of immunotherapies with immune checkpoint inhibition and cell therapies, revolutionising how one’s cancer is now treated [238]. Despite this, chemotherapy remains a first-line treatment for many cancers and is recognised as an effective co-therapy with immune checkpoint inhibition for preventing tumour progression [239]. While effective, many of the patients fortunate enough to reap the benefits of such treatment often progress to having equally morbid cardiovascular complications. Hence, the use of polyphenols to mitigate and even enhance such therapies is warranted.

Polyphenols have previously been shown to improve the efficacy of checkpoint inhibition by downregulating key receptors and ligands such as PD-1 and PDL-1 [240,241]. In fact, the use of polyphenols as adjuncts in immunotherapy have already been extensively reviewed by others [242,243,244]. Numerous clinical trials have been performed to try to utilise the ability of these organic molecules to enhance drug efficacy and reduce chemoresistance. In fact, in a cohort of patients with inoperable colorectal cancer, a recent clinical trial (NCT01490996) assessed the safety and tolerability of curcumin (≤2 g) administered together with a 5-FU and OXA (FOLFOX) cocktail [245]. Compared to patients treated with FOLFOX alone, subjects provided with the polyphenol–chemotherapy cocktail displayed higher overall survival and longer median progression-free survival [245]. More recently, patients with advanced gastric cancer were treated with a resveratrol and copper mixture in combination with a docetaxel-based triplet chemotherapy (CTRI/2019/07/020289) [246]. Notable improvements in patient outcomes included marked reductions in non-haematological side effects commonly associated with chemotherapy, such as diarrhoea and vomiting. Such studies provide a reason to be optimistic about the translational potential of these compounds as novel components in future anti-cancer treatments.

## 6. Conclusions

Multiple preclinical sources and now newly emerging clinical evidence suggest that polyphenols exert vasoprotective effects. Thus, the utilisation of these organic molecules represents an attractive strategy for counteracting chemotherapy-induced endothelial dysfunction (ED). To date, several of these compounds have not achieved clinical success due to their aforementioned limitations. However, as nanotechnology continues to evolve, many of these barriers are beginning to diminish, paving the way for future therapeutic applications. Whether polyphenols can significantly enhance the efficacy of anti-cancer treatments remains to be determined; nonetheless, their potential as adjuncts to protect the vascular endothelium is supported by an encouraging body of evidence and warrants further investigation. Moving forward, their integration into cancer care should be guided by studies that utilise validated vascular endpoints—such as the flow-mediated dilation (FMD), endothelial biomarkers, and vascular imaging—while also considering advanced delivery platforms like nanoparticle encapsulation, which may overcome current bioavailability challenges and accelerate clinical translation.

## Figures and Tables

**Table 1:** Common chemotherapy drugs and the types of cancers they are licenced to treat according to the US Food and Drug Administration.

Chemotherapy Class	Drug	Indications	Source
Anthracyclines	Doxorubicin	Acute leukaemias; Hodgkin’s lymphoma; non-Hodgkin’s lymphoma; solid tumours (including breast cancer); soft tissue sarcoma; bladder cancer; AIDS-related Kaposi’s sarcoma; ovarian cancer; multiple myeloma	[14]
	Epirubicin	Breast cancer; gastric cancer; small cell lung cancer; ovarian cancer; colorectal cancer; lymphoma; leukaemia; multiple myeloma; bladder cancer	[15]
Non-anthracycline antitumour antibiotics	Bleomycin	Squamous cell carcinoma of the head and neck, cervix, and external genitalia; testicular cancer; non-Hodgkin’s lymphoma; Hodgkin’s lymphoma	[16]
Alkylating agents	Cisplatin	Testicular cancer; ovarian cancer; lung cancer; bladder cancer; squamous cell carcinoma of the head and neck; cervical carcinoma	[17]
Antimetabolites	Fluorouracil	Solid tumours (including gastrointestinal tract and breast cancers); colorectal cancer; superficial malignant and premalignant skin lesions	[18]
Topoisomerase inhibitors	Etoposide	Testicular cancer; small cell lung cancer; Hodgkin’s lymphoma; non-Hodgkin’s lymphoma; acute myeloid leukaemia; ovarian cancer; gestational trophoblastic neoplasia	[19]
Mitotic inhibitors	Docetaxel	Breast cancer; non-small cell lung cancer; prostate cancer; gastric adenocarcinoma; squamous cell carcinoma of the head and neck	[20]

## Data Availability

No new data were created or analysed in this study. Data sharing is not applicable to this article.
